# Infection of the fracture hematoma from skeletal traction in an asymptomatic HIV-positive patient: Additional support for early surgical treatment of femoral fractures in people living with HIV in low-income countries?

**DOI:** 10.3109/17453674.2012.704564

**Published:** 2012-08-25

**Authors:** Sven Young, Fletcher J Beniyasi, Boston Munthali, Leonard Banza

**Affiliations:** ^1^Department of Surgery, Kamuzu Central Hospital, Lilongwe, Malawi; ^2^Department of Orthopedic Surgery, Haukeland University Hospital; ^3^Department of Surgical Sciences, University of Bergen, Bergen, Norway

In June, 2011, a 27-year-old man was walking home in the dark, in Lilongwe, Malawi, when he fell several meters into an open septic tank that was under construction. He was brought to Kamuzu Central Hospital (KCH), the main government referral hospital for the central region of Malawi, in considerable pain and was found to have sustained a left closed subtrochanteric spiral fracture (Figure 1). He also sustained a brachial plexus injury on his left side with complete loss of function of the shoulder and elbow, but with some hand function preserved. Hemoglobin test was not done on admission due to the full blood count (FBC) machine in the laboratory having broken down. As would be the case for femoral fractures in most hospitals in the region, proximal tibial skeletal traction was applied. The brachial plexus injury was treated with a sling only.

**Figure 1.**
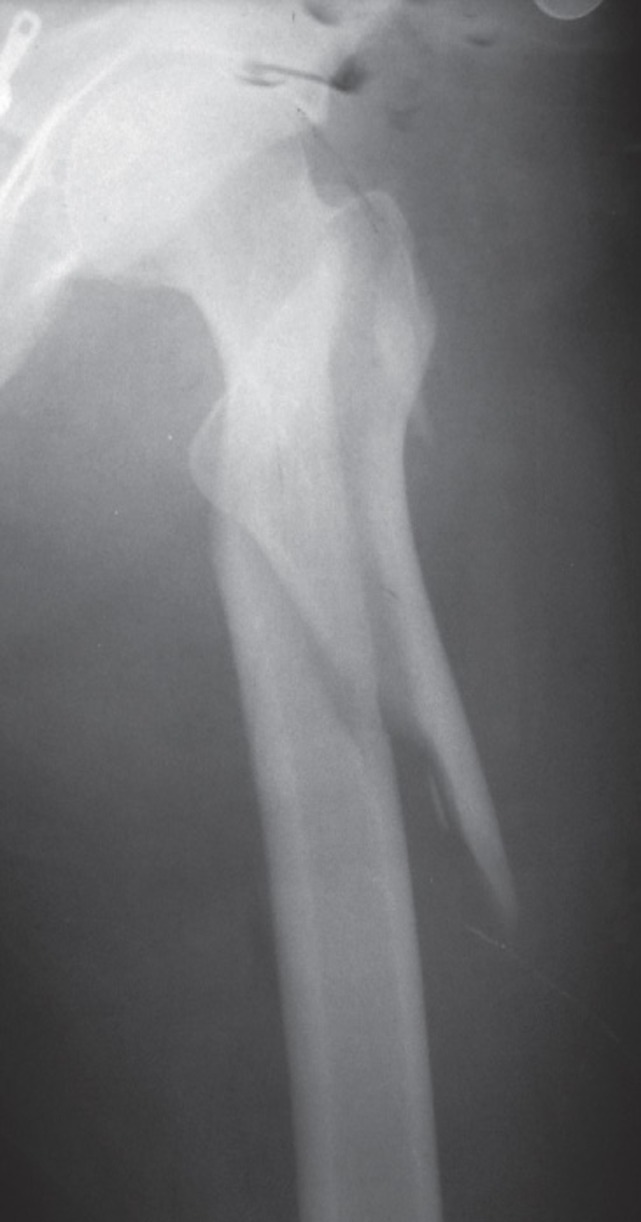
The day after admission to hospital. At KCH, more often than not, the lack of films and chemicals prevents getting a second view.

The patient was HIV-positive and had started antiretroviral treatment (ART) only a few weeks before the accident. A CD4 count result was not available to us, but in Malawi ART is started only when the CD4 count is below 350. He had no AIDS-defining conditions, had not lost weight, had no symptoms or complaints from his HIV infection, and as such was probably WHO clinical stage 1 (WHO 2007) and CDC class A2 (CDC 1992). He was admitted to the male orthopedic ward on traction. This ward is designed for 40 beds, but at any time has between 60 and 90 inpatients, and most of the time there is only one qualified nurse on duty. KCH has surgeons who are trained to do locked intramedullary (IM) nailing of fractures, and it has the equipment. A successful operation with an IM nail would have led to this patient being out of bed in a few days, but because of a heavy workload, a severe lack of staff and theater time, and the fact that this fracture was considered by the treating clinicians to have good potential for of healing, it was decided to continue treatment with traction until union.

We saw the patient 3 months later. He had spent the entire 3 months on traction in bed without physiotherapy or exercises. A new radiograph showed increased angulation at the fracture site and no obvious callus (Figure 2). His knee was stiff in extension with only 20 degrees range of motion. The fracture was still mobile and very painful on manipulation. There was a pin-site infection with a little pus discharging from the medial side of the traction pin, but no tenderness over the soft tissues around the pin sites and knee. The patient was in a great deal of discomfort and had already been away from his family and work for 3 months. Because of this, he was counseled on the benefits and risks of IM nailing, and he agreed readily to having surgery. The traction pin was removed and the infected wound washed and dressed for 2 weeks until the pin sites were clean and dry. During this time, the patient’s pain increased. However, this was interpreted as pain because of increased mobility of the fracture after the traction was removed. Before surgery his hemoglobin was 9.0, white blood cell count 8.1, platelet count 369, and his CD4 count was 295.

**Figure 2.**
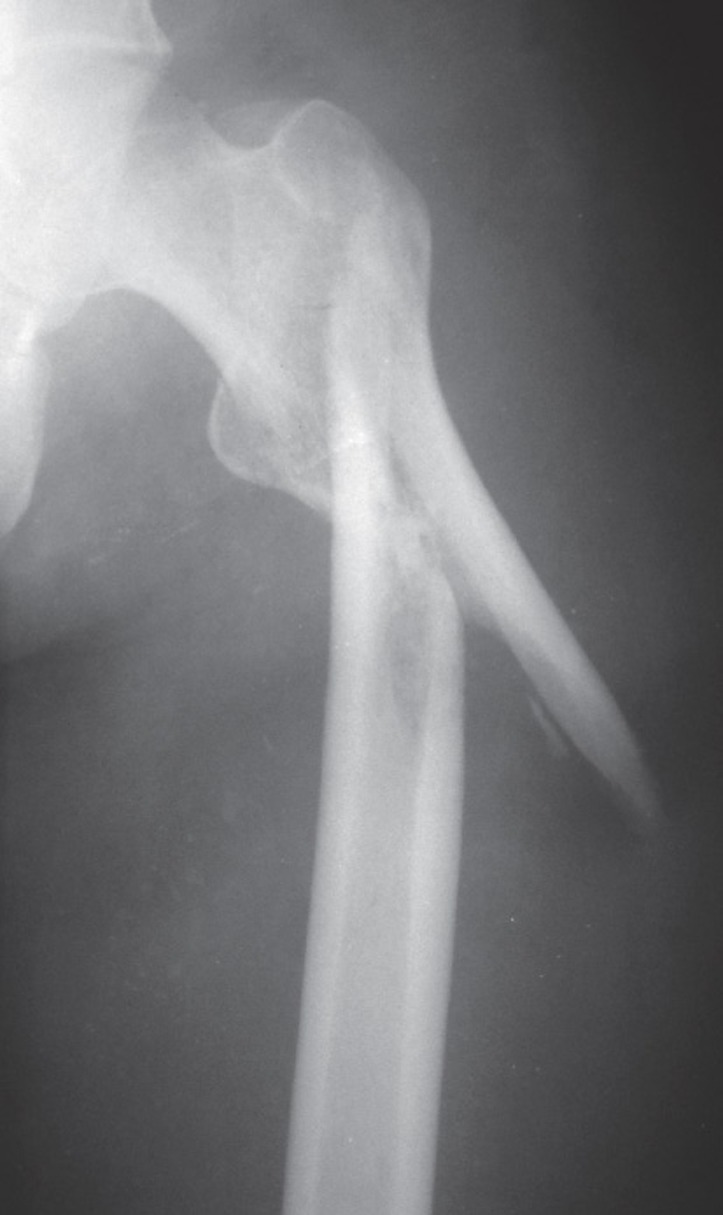
After 3 months on skeletal traction. Yet again, a lateral radiograph was not possible because of lack of resources. There was no obvious callus formation. There was some rounding of the sharp fragment ends and the displacement had increased.

On the day of surgery the patient’s thigh was still a little swollen, but the skin was intact. There was no rubor or increased warmth in the thigh. However, as soon as the fascia was incised, a large amount of pus drained into the wound. Approximately 300 mL of pus was drained from an extensive cavity between the fascia and the quadriceps muscles on the proximal thigh. No communication was found extending to the level of the knee. The cavity was thoroughly irrigated and mechanically cleaned. A drain was left in the cavity and the wound loosely adapted. Naturally, the planned IM nail was not inserted. Pus was sent for culture and sensitivity, but results were never received, as the lab had run out of supplies.

Postoperatively, the patient was treated with intravenous Ceftriaxone (1g once daily). The drain was removed after 2 days. 10 days postoperatively, the hospital ran out of Ceftriaxone. Treatment was continued with the only 2 available antibiotics at that time apart, from benzyl-penicillin: Cefalexin tablets (500 mg 4 times daily) and intravenous Gentamycin (240 mg once daily). The infection settled surprisingly quickly. The patient was in less pain on the first day after surgery and continued to improve steadily over the next few weeks. He was mobilized on crutches 11 days postoperatively and was discharged 22 days after surgery. Oral Cefalexin was prescribed for 2 more weeks after discharge. Despite getting his phone number and address and repeatedly trying to contact him, we never saw the patient again.

## Discussion

An estimated 5.8 million people die of injuries each year, 32% more than the number of fatalities that result from malaria, tuberculosis, and HIV/AIDS combined (Mathers et al. 2008). Over 90% of deaths due to injuries occur in low- and middle-income countries (LMICs). For every patient that dies as a result of injuries, many more survive with permanent disability (Peden 2004). Although Africa has 24% of the global burden of disease, it has only 3% of health workers and commands less than 1% of world health expenditure (WHO 2006). Of the estimated 234 million major surgical operations performed in 2010, only 3.5% were performed in the poorest 35% of the world population (Weiser et al. 2008).

Even the most basic resources in hospitals in high-income countries such as simple blood tests and plain radiographs are scarce in many hospitals in LMICs. In these countries, the modest amount of attention surgically treated disease has received has been focused on district hospitals. However, as can be seen from our case, even the central referral hospital in the capital city of Malawi, with a catchment population of 5.5 million people, does not have stable access to these basic services.

Treatment of femoral fractures in adults with traction involves hospitalization for at least 6 weeks, and often a great deal longer. Contrary to many surgeons’ beliefs, treatment of femoral fractures in adults with traction is actually resource-intensive (Gosselin and Lavaly 2007). It requires constant vigilance to avoid malunion, joint contractures, and deep vein thrombosis (DVT). The reality in many hospitals in LMICs is, however, that the human resources to take care of this are not available. As a consequence, patients—like our patient—are left to fend for themselves in the ward with the help of family members. Invariably, this ends with a stiff knee and often with shortening and external rotation of the fracture. Close to half of all skeletal traction patients get pin-tract infections (Gosselin and Lavaly 2007).

An increasing body of research is showing that internal fixation of femoral fractures is as safe in LMICs as it is in high-income countries (Saris et al. 2006, Gross et al. 2010, Sekimpi et al. 2011, Young et al. 2011), where it is generally considered the only ethical option. Even the argument that this kind of treatment is too expensive to merit support in LMICs is losing ground, as new research is increasingly showing that surgery is cost-effective even in low-resource settings (Laxminarayan et al. 2006, Gosselin et al. 2009).

In addition to the resource constraints and the increased burden of injuries facing LMICs, many countries have the added burden of a high prevalence of HIV. HIV patients have a moderately increased risk of complications, including wound infection (Paiement et al. 1994), especially after open fractures. However, it seems that the degree of contamination of the wound in open injuries (Guth et al. 1996, Harrison et al. 2002) and the lifestyle of the patients (Habermann et al. 2008) have more influence on outcome than HIV infection alone. There is increasing evidence both from high-income countries and from LMICs that the end outcomes after surgery in general are no worse for patients with HIV than for other patients (Cacala et al. 2006, Duane et al. 2008, Stock et al. 2010, Patel et al. 2011).

Our patient was hospitalized for 4 months, resulting in considerable personal and economic loss. The development of an infected fracture hematoma also deprived him of delayed surgery. The modest findings preoperatively delayed the diagnosis and were probably the result of his immunodeficiency. In our opinion, the most likely source of his infection was the infected pin site, especially since the increasing pain from the thigh came shortly after the pin-site infection. No evidence of a sub-fascial route for the spread of this infection could be found. There may therefore have been hematogenous seeding to the fracture hematoma facilitated by his immunodeficiency. Of course, one cannot rule out other sources of hematogenous infection. Pyomyositis in patients with HIV is also well known without any injury or traction. However, the patient did not have other known infections or symptoms. The authors have also seen one other case of infected fracture haematoma in a patient on skeletal traction at KCH. This patient was HIV-negative, but severely malnourished and possibly immunosuppressed as a result of this. Even if the source of infection was not the pin-site infection, these cases still show the possibility of infection of a femoral fracture while on traction. While identical bacterial cultures from the pin site and fracture would have increased the likelihood of the pin site being the source, only a large prospective study could actually quantify this risk and show an increased risk for HIV patients. There is, however, enough compelling evidence in the literature already to support the idea that HIV infection is not a contraindication to IM nailing of femoral fractures.

Follow-up is a continuous problem in Malawi, as in many other low-income countries. Most patients do not return for review—even if actively encouraged—unless they have a serious problem. The most frequent reason given is the cost of transport. Our patient is hopefully still alive, but if so he most probably still has a painful non-united femoral fracture and a stiff knee.

In conclusion, this case illustrates the fact that the treatment of femoral fractures in adults with traction is not without complications, and that there may be an added risk for people living with HIV. Immunodeficiency increases the risk of infection and at the same time it reduces the clinical signs of infection, thus delaying diagnosis. There is growing evidence that IM nailing is a safe and cost-effective procedure also in low- and middle-income countries when carried out for the right indications and by well-trained surgeons, and that early internal fixation of closed fractures in HIV patients is safe. In light of this, there is increasing support for the use of early intramedullary nailing of femoral fractures in HIV-positive patients also in low-income countries.
